# Tumor Microenvironment in Neuroblastoma and Immunotherapeutic Approaches: Towards More Effective Treatment

**DOI:** 10.3390/cancers18071079

**Published:** 2026-03-26

**Authors:** Irina Zh. Shubina, Chi-Bao Bui, Truc Ly Nguyen, Anatoly P. Kazantsev, Duy Khang Nguyen, Quynh Giang Nguyen, Khang Thinh Tran, Natalya A. Burlaka, Nikolay Yu. Sokolov, Kirill I. Kirgizov, Svetlana R. Varfolomeeva, Mikhail V. Kiselevskiy

**Affiliations:** 1 FSBI “N.N. Blokhin National Medical Research Center of Oncology” of the Ministry of Health of Russia, Kashirskoye sh. 24, Moscow 115522, Russia; oncoanat@mail.ru (A.P.K.); dreamfull2009@yandex.ru (N.A.B.); k.kirgizov@ronc.ru (K.I.K.); s.varfolomeeva@ronc.ru (S.R.V.); kisele@inbox.ru (M.V.K.); 2 Department of Biochemistry and Immunology, University of Health Sciences, Vietnam National University Ho Chi Minh City, Ho Chi Minh City 700000, Vietnam; nttly@uhsvnu.edu.vn (T.L.N.); ndkhang.y2021@uhsvnu.edu.vn (D.K.N.); nqgiang.y2019@uhsvnu.edu.vn (Q.G.N.); tkthinh.y2023@uhsvnu.edu.vn (K.T.T.); 3 SBHI S.P. Botkin Hospital, 2nd Botkinsky pr-d, 5, Moscow 125284, Russia; strivp@mail.ru

**Keywords:** neuroblastoma, high-risk, tumor microenvironment, autologous stem cell transplantation, immunotherapy, chimeric antigen receptor T cells, bioinformatics, neuro-antigens

## Abstract

**Background/Objectives**: High-risk neuroblastoma (HR-NB) is a major cause of cancer-related death among children. The review aims to discuss various biochemical and genetic traits of neuroblastoma (NB) used for the potential of cell-based therapies. **Methods**: A comprehensive search was performed through MEDLINE, PubMed, Scopus, and ScienceDirect using various combinations of “neuroblastoma”, “tumor microenvironment (TME)”, “immune cells”, “non-immune cells”, “hematopoietic stem cell transplantation (HSCT)”, “autologous stem cell transplantation (ASCT)”, “natural killer cells (NK)”, “chimeric antigen receptor T cells (CAR-T)”, “CAR-NKT”, “tumor infiltrating lymphocytes (TIL)”, “bioinformatics”, and “neuro-antigens” in the published papers over the last decade. Reviews, systematic reviews, and clinical trials related to children’s NB were selected. The final set included 106 articles of interest. **Results**: Recent studies have shown that TME is crucial in determining the malignancy, immune evasion, and drug resistance of NB. Innate immune or non-immune cells play important roles in shaping the NB TME. Depleting or reprogramming TME factors can improve the effectiveness of immunotherapy. A number of clinical trials have studied and showed feasibility of using ASCT, NK cells, CAR-T, and CAR-NKT cells in the adoptive therapy for HR-NB. However, an unambiguous evaluation of the effectiveness of cell-based technologies in the HR-NB therapy is still complicated due to the lack of large randomized trials. **Conclusions**: The reported small and non-randomized studies that demonstrated controversial results cannot prove, undoubtedly, the promising potential of the cell-based technologies including ASCT, NKs, CAR-T, and CAR-NKT cells. Further randomized clinical trials, using the same treatment, will help determine the role in the multimodal treatment for HR-NB.

## Simple Summary

Neuroblastoma is the most common extracranial solid tumor in young patients. High-risk neuroblastoma still remains a major cause of cancer-related death among children. The standard treatment is provided according to the risk assessment, which is based on clinical, biochemical and molecular genetic factors. Over the last years, innovative technologies and cell-based therapies, including autologous stem cell transplantation and CAR-T cell immunotherapy, have shown their potential for high-risk neuroblastoma. Recent studies have been aimed at the integration of artificial intelligence for diagnostic and therapeutic purposes. This review presents an up-to-date view on neuroblastoma biology, current schemes of the effective therapy and future potential of innovative technologies.

## 1. Introduction

### 1.1. Overview of Neuroblastoma

Neuroblastoma (NB) is the most common extracranial solid tumor in young patients [1]. It accounts for 8–10% of all juvenile malignancies, yet it is the main cause of cancer-related death in this age range, particularly among children under the age of five [2]. NB usually begins in the abdomen (frequently in the adrenal glands), but it can also start in the chest, neck, or pelvis, which correlates to sympathetic nervous system locations. The condition is caused by neural crest cells, a flexible cell population that forms throughout embryogenesis and serves as a precursor for many structures in the peripheral nervous system, including the sympathetic nervous system [1,3]. NB tumors can emerge as a result of abnormal cell development and differentiation. NB’s clinical presentation and disease course are highly heterogeneous, making it a unique and serious problem. Some tumors can regress spontaneously without therapy, especially in newborns, a condition called spontaneous regression [1]. In contrast, high-risk NB (HR-NB) cases tend to spread significantly, display treatment resistance, and have a dismal prognosis despite vigorous therapy [4,5]. Genetic and molecular variables have a significant impact on disease prognosis. For example, *MYCN* gene amplification is typically associated with severe illness, rapid disease progression, and a poor prognosis. Furthermore, DNA methylation changes, deletions of chromosomal 1p or 11q, and gains of chromosome 17q are associated with high-risk disease [5]. Understanding NB’s various biochemical and genetic traits is critical for risk assessment, allowing for personalized treatment regimens ranging from observation to chemotherapy, surgery, radiation, and bone marrow transplantation [3,5]. Advances in precision medicine and genomic research are paving the door for new therapeutic approaches to this complex disease.

### 1.2. Tumor Microenvironment: A Critical Factor

For many years, research on NB concentrated on intrinsic tumor factors such as genetic alterations, *MYCN* amplification, and chromosomal abnormalities. However, new research suggests that the tumor microenvironment (TME) is crucial in determining the malignancy, immune evasion, and treatment resistance of NB [3,4]. The TME is made up of different non-cancerous components such as immune cells (macrophages and T cells), blood vessels, fibroblasts, the extracellular matrix (ECM), and cytokines. These components have complex interactions with tumor cells, which promote tumor growth and metastatic dissemination [3,4]. Notably, the prevalence of tumor-associated macrophages (TAM) and immunosuppressive cells such as regulatory T cells (Treg) and myeloid-derived suppressor cells (MDSC) in NB has been linked to poor prognosis and resistance to immunotherapy [4]. Therapeutic methods have recently evolved to focus on the TME, such as restoring immune responses by combining checkpoint inhibitors with innate immune activators or blocking TAMs to undermine the tumor’s protective barrier [4]. These techniques show potential for enhancing treatment efficacy in NB, especially for patients with “cold” and treatment-resistant lesions [6,7,8].

#### 1.2.1. Immune Cells

The NB TME is a complex ecosystem of immune and non-immune cells that significantly influences tumor progression, immune evasion, and therapeutic prognosis/outcomes. Immune cells within the TME are categorized into innate and adaptive immune cells, each with distinct roles in modulating tumor behavior. Recent studies leveraging single-cell transcriptomics and computational algorithms have elucidated their functions, mechanisms, and novel therapeutic implications [7,9].

Innate immune cells, including TAMs, natural killer (NK) cells, dendritic cells (DC), and MDSCs play critical roles in shaping the NB TME. TAMs are a dominant population, particularly in high-risk, *MYCN*-amplified tumors, where they often adopt an M2-like immunosuppressive phenotype [10]. TAMs function together with cancer-associated fibroblasts (CAF) to promote tumor growth and immune evasion by secreting immunosuppressive cytokines [11]. These cytokines inhibit cytotoxic immune responses and enhance tumor cell survival. Also, TAMs’ role in chemoresistance emphasizes their production of sIL-6R, which promotes CAF-mediated STAT3 signaling in tumor cells, encouraging drug resistance [12]. Moreover, TAMs in NB induce hypoxia-inducible factor 2α (HIF-2α) production, fostering a hypoxic TME that inhibits NK cells and supports tumor progression [13]. As a result, high TAM infiltration correlates with poor prognosis in *MYCN*-amplified tumors [10], and boosts vascular endothelial growth factor (VEGF) production as it is a downstream target of HIF2α [14]. Therapeutic strategies targeting TAMs, such as CSF1R inhibitors, show promise in reprogramming TAMs toward an M1-like antitumor phenotype [4].

Another essential innate component, NK cells, have strong cytotoxic effects on NB cells, especially those that express low MHC class I, which is a characteristic of NB. However, studies showed that TAM-derived tumor growth factor (TGF)-β and IL-10 suppress NK cell activity, reducing their cytotoxic potential [15,16]. Adoptive NK cell therapies or the use of anti-GD2 antibodies or an immune-modulating drug that activates IL-2 secreting T cells (e.g., lenalidomide) to improve NK function can overcome TME-mediated suppression [6,15]. DCs, though less abundant, are crucial for antigen presentation and associated with a better prognosis, but are often functionally impaired in the TME due to TAM- and tumor-derived immunosuppressive signals [17]. Promising clinical strategies related to DCs include anti-PD-1/PD-L1 therapies increasing CD11c+ MHC II+ DC infiltration in murine NB models, thereby enhancing antigen presentation and synergizing with T cell responses [18] or the DCs vaccines [19]. MDSCs, particularly polymorphonuclear (PMN), contribute to immunosuppression by generating reactive oxygen species and arginase-1, or producing IL-10 and TGF-β, limiting T and NK cell activities [4,13] and Tregs [20]. The identification of LOX-1 and CD84 as particular markers for PMN-MDSCs allows them to be distinguished from neutrophils and provides opportunities for targeted depletion techniques employing anti-LOX-1 therapeutics [13]. These results demonstrate how immunosuppressive innate cells may be depleted or reprogrammed to increase the effectiveness of immunotherapy.

**Figure 1:**
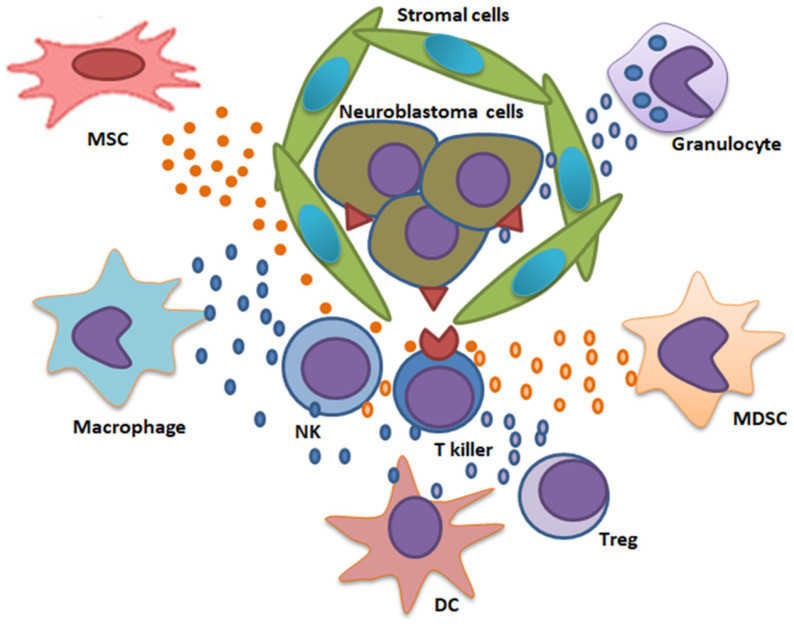
Microenvironment of the neuroblastoma cells including immune suppressor cells. MSC—mesenchymal stem cells; NK—natural killer cells; DC—dendritic cells; MDSC—myeloid-derived suppressor cells; T killer—cytotoxic T cells; T reg—T regulatory cells.

Adaptive immune cells, primarily T cells and B cells, are pivotal in mounting specific antitumor responses but face significant challenges in the NB TME. T cells, including cytotoxic CD8+ T cells and Tregs, exhibit heterogeneous infiltration patterns. *MYCN*-non-amplified tumors display higher cytotoxic T cell infiltration, correlating with an inflammatory, “hot” TME and better survival outcomes. Conversely, *MYCN*-amplified tumors are “cold,” with low T cell infiltration due to immunosuppressive signals from TAMs and Tregs [6]. Tregs are identified as a key suppressor, via IL-10 and TGF-β production, inhibiting antigen-presenting cell maturation to dampen T cell activity [20]. A significant insight from recent studies is the prognostic value of immune cell composition, which developed a prognostic cell risk score that incorporates T cell and Treg abundance to predict patient outcomes, validated across multiple datasets [9]. B cells, though less studied, contribute to the TME through antibody production and antigen presentation. However, this suggestion is still controversial: on the one hand, a study showed that B cell infiltration is higher in the low-risk but absent in the high-risk NB [6], while another study found a significant increase in memory B cells in the intermediate- and high-risk disease compared with the low-risk tumors [21]. Like TAMs, MDSCs produce multiple immunosuppressive factors. They proliferate and increase in numbers as the tumor progresses and play a crucial role in tumor development, metastasis, and resistance to treatment. MDSCs are a heterogeneous population of immune cells, which contribute to the immunosuppression of the TME via direct and indirect modulation of other immune cells, such as T cells, NK cells, antigen-presenting cells, DCs and B cells, and may stimulate tumor development through the secretion of factors such as reactive oxygen species, Arg-1, and TGF-β [22,23]. The stroma surrounding NB cells makes a mechanical barrier that prevents direct interaction of effector lymphocytes (T killers and NKs) with tumor cells. MSCs with tollerogenic properties produce suppressor factors, in particular, TGF-β. MDSCs producing a range of cytokines have a suppressive effect on the immune cell functions. In addition, T regulatory cells (T regs) may have their impact in immune cell suppression (Figure 1).

#### 1.2.2. Non-Immune Cells and Components

CAFs make up the most abundant stromal component in the TME and are characterized by the expression of alpha-smooth muscle actin (αSMA) and fibroblast activation protein (FAP). They facilitate tumor growth and the subsequent spread of the tumor to other parts of the body by remodeling the ECM and by the secretion of growth factors such as VEGF and TGF-β [1,6,19]. CAFs have also been found to promote metastasis by increasing angiogenesis through VEGF release and changing the ECM via collagen synthesis [6]. They also interact with tumor and immune cells, including TAM. Together, they promote a more immunosuppressive TME by producing inflammatory cytokines and TGF-β, which inhibit immune response and enhance tumor cell survival [11,19]. Hashimoto et al. showed that CAFs significantly correlate with more aggressive features of NB, such as *MYCN* amplification (*p* = 0.045) and more advanced clinical stage (*p* = 0.001), and bone marrow metastasis (*p* = 0.009). They performed immunohistochemistry to quantify αSMA-positive CAFs, deducting h-caldesmon-positive areas to identify CAFs rather than vascular smooth muscle cells, and showed their association with TAMs, implying synergistic interactions. The NB-conditioned medium was able to activate CAF-like BM-MSCs by upregulating αSMA and FAP expression in a time-dependent manner, indicating stromal cell activation [11]. Another novel finding highlights that CAFs in *MYCN*-A tumors secrete pro-inflammatory lipid mediators, such as prostaglandin E2, which enhance TAM infiltration and tumor growth [19].

Endothelial cells make up the tumor vasculature, which facilitates nutrition and oxygen supply to promote tumor development and spread. In NB, endothelial cells are stimulated by VEGF and other angiogenic factors secreted by tumor cells, CAFs, and TAMs, which promote angiogenesis and vascular mimicry [3,11]. Noguera et al. discovered that high HIF-2α protein levels in NB (due to high TAMs infiltration) are linked to poor outcomes because HIF-1α and HIF-2α can transcribe VEGF in hypoxic conditions, as demonstrated in vitro and in vivo, leading to vascular formation. Anti-angiogenic therapies, such as bevacizumab, could disrupt this process, though resistance remains a challenge due to CAF- and TAM-derived compensatory signals. The NANT consortium has completed a Phase I trial targeting endothelial cells in NB with bevacizumab, cyclophosphamide, and zoledronic acid in patients with recurrent or refractory HR-NB (NCT00885326) [3].

Schwannian stromal cells are specific to NB and are associated with less aggressive phenotypes such as ganglioneuroblastomas. The International Neuroblastoma Pathology Committee divides NB into diagnostic groups depending on the quantity of Schwannian stromal cells, emphasizing the significance of this component [24]. They release neurotrophic substances (e.g., nerve growth factor), which promote tumor cell differentiation and suppress proliferation, resulting in spontaneous regression in low-risk tumors. [3]. Zeine et al. [25] discovered an adverse association between Schwannian stromal cell populations and CAF abundance, with high CAF concentration associated with poorly differentiated and high-risk malignancies. This suggests that Schwannian cells may counteract CAF-driven tumor progression.

Collagen is a key ECM component in the NB TME, functioning not only as a structural scaffold but also as an active regulator of tumor progression. COL11A1—a gene providing instructions for making a component of type XI collagen—is shown to be related to and upregulated by CAFs [26]. High COL11A1 expression is correlated with advanced stage, recurrence, undifferentiated histology, and poor prognosis, and its silencing lowers NB invasiveness, highlighting it as a potential therapeutic target [26,27]. Beyond structural roles, collagen influences tumor cell behavior through mechanotransduction; increased ECM stiffness and collagen alignment activate integrin-FAK and downstream PI3K/AKT/MAPK signaling, promoting epithelial–mesenchymal transition, while certain collagen contexts can enhance neuronal differentiation and reduce *N-Myc* expression [28,29]. Recent advances in 3D collagen-based NB models have recapitulated in vivo architecture, ECM–cell interactions, and chemoresistance patterns, providing powerful platforms for studying ECM-driven tumor biology and testing therapies that target collagen synthesis, crosslinking, or signaling [30,31,32]. Moreover, thermal ablation has been applied in NB experiments to discover its impact on collagen and TME [32,33]. Studies showed that thermal ablation not only is more effective in destroying NB cells, compared to cryo-ablation but also could permanently break down collagen structure in TME and alter the immune cells, components in a positive way, especially in stage III-IV NB [34]. Together, these findings underscore collagen’s multifaceted role in NB pathobiology and its promise as both a biomarker and therapeutic target.

## 2. Current Therapy

### 2.1. Search Strategy for the Relevant Therapy Reports

**Figure 2:**
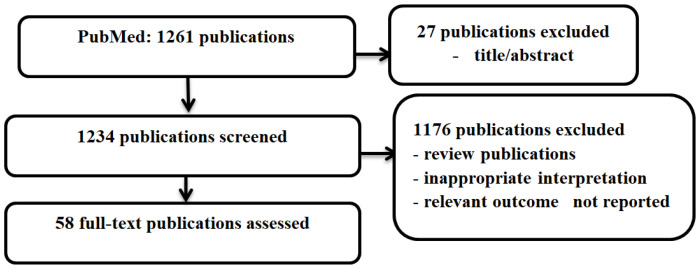
Search strategy for including recent therapy reports for the analysis.

To evaluate the available HB-NB treatment approaches, a systematic search was performed in the electronic databases of MEDLINE through PubMed. The search strategy included randomized, non-randomized, and pilot studies. The literature search yielded 1261 results; the final set resulted in 58 publications with the reports of clinical trials included in the review of current therapy for HR-NB (Figure 2). Of these, seven papers included information on four randomized controlled trials (RCT), and the others referred to non-RCT studies. The studies were different in terms of the populations included, induction therapy regimens, and responses to the induction therapy, additional therapies, and transplantation procedures.

At present, NB is considered as one of the most difficult childhood malignancies to cure, especially in high-risk populations. Current treatments often include surgery, chemotherapy, radiation, stem cell transplantation, and immunotherapy [4].

Surgery remains the primary treatment option for localized malignancies, particularly when total resection can be achieved. However, in cases of metastatic or high-risk cancer, surgery has a limited role and is frequently added by systemic therapy. Chemotherapy is a critical component of NB treatment, especially during the induction and consolidation phases. Cyclophosphamide, doxorubicin, vincristine, cisplatin, and etoposide are all commonly utilized medicines. Due to tumor cell chemoresistance, patients frequently require high-dose chemotherapy followed by autologous stem cell transplantation to restore bone marrow function [4]. Radiotherapy is used to treat residual disease following surgery or chemotherapy, usually focusing on the main tumor site. Furthermore, systemic irradiation using meta-iodobenzylguanidine paired with radioactive iodine is increasingly used and has demonstrated great benefit in patients with recurrent diseases [35].

### 2.2. Anti-GD2 Immunotherapy

Immunotherapy has emerged as a significant breakthrough in the last decade. The FDA has approved monoclonal antibodies for high-risk NB (HR-NB), including dinutuximab (Unituxin) and, more recently, naxitamab (Danyelza), which target the GD2 antigen expressed on the surface of the NB cells. According to the National Cancer Institute’s 2022 report, these medicines considerably increase progression-free and overall survival in children with HR-NB [4,36]. However, not all patients react to anti-GD2 treatment; about 50% of patients develop relapses due to limited antibody penetration, immune resistance, or potential antigen loss (GD2-negative/low), especially in relapsed or refractory high-risk diseases [8]. Ongoing research aims to improve efficacy by combining these treatments with IL-2 or GM-CSF or integrating immunotherapy with checkpoint inhibitors, but their impact in NB treatment remains limited due to its “immunologically cold” tumor features. Most of the studies that involved patients with relapse/refractory (R/R) NB used a 5-cycle anti-GD2 therapy. Although some studies reported 6–10 cycles, no evidence of the improved survival was provided that could show the benefit of the increased number of cycles of the anti-GD2 therapy [37].

The first-line therapy does not require confirmation of high level of GD2 expression on the neuroblastoma cells [38]. Usually, GD2 expression on the NB cells is well-detected during the anti-GD2 treatment; however, some authors reported that the antigen shedding could occur in course of the therapy [39]. Obviously, this mechanism of avoiding the anti-GD2 antibody exposure contributes to the decreased effectiveness of the increased number of dinutuximab beta (DB) cycles. In addition, it was shown that neutralizing antibodies might be produced in response to anti-GD2 antibody treatment, which could also reduce the clinical effectiveness of anti-GD2 treatment, although there is no evidence of a negative effect on anti-GD2 antibodies [40].

The toxicity of DB in the first-line maintenance therapy has been well studied, and the side effects of this therapy are manageable [41]. The toxicity profile described in the R/R HR-NB clinical trial was comparable to the profile observed in the first-line therapy. Low doses of inteleukin-2 (IL-2) were used to improve the effectiveness of the anti-GD2 therapy. However, the combination of DB with IL-2 in patients with HR-NB and R/R NB did not reveal the advantages of the combination compared with the DB monotherapy [42].

Additionally, cellular therapies such as anti-GD2 CAR-T cell therapy are being studied in preclinical and early-phase clinical trials. In vitro and in vivo experiments have shown that GD2-targeted CAR-T cells can destroy NB cells, although problems such as toxicity and tumor evasion still remain unresolved [4].

### 2.3. HSC Transplantation

Allogeneic hematopoietic stem cell transplantation is an integral stage of therapy, demonstrating high efficacy in patients with hematological malignancies, especially in high-risk patients [43]. On the other hand, autologous stem cell transplantation (ASCT) is not the standard treatment for a number of high-risk extracranial solid tumors. Moreover, several studies reported that high-dose chemotherapy in combination with ASCT in different types of sarcomas, Wilms tumors, and hepatoblastomas did not significantly increase the relapse-free survival and overall survival (OS) [44,45,46,47]. Unlike other solid tumors, the results of high-dose chemotherapy in combination with ASCT for patients with HR-NB were more optimistic. In particular, tandem ASCT after high-dose chemotherapy for patients with HR-NB improved the treatment effectiveness. A large randomized clinical trial involving 652 patients with HR-NB demonstrated that tandem ASCT significantly improved event-free survival (EFS) (61.6% vs. 48.4%) compared with single ASCT, though OS did not differ significantly in those groups [48]. Another randomized trial recruited 539 patients aged 1 to 18 years with newly diagnosed HR-NB. This clinical study compared myeloablative chemotherapy and radiation therapy combined with ASCT with continuing intensive chemotherapy without ASCT. The myeloablative therapy and ASCT resulted in a significantly beneficial 5-year progression-free survival and overall survival as compared to the non-myeloablative chemotherapy [49].

An early randomized trial evaluated the effectiveness of a conditioning regimen followed by infusion of autologous purged bone marrow transplantation (ABMT) in comparison with the chemotherapy alone. The study did not show a significant improvement in OS in the ABMT group [50]. Similarly, in 2013, a Cochrane meta-analysis found no improvement in OS in patients with HR-NB after ABMT/ASCT. A later analysis conducted by the SIOPEN group showed that ABMT in patients with HR-NB led to a slightly positive effect on EFS [51]. To date, the question whether to perform single or tandem ASCT in patients with HR-NB remains unresolved; since even the clinical trials that showed effectiveness, did not achieve OS improvement, and they demonstrated effectiveness only at surrogate endpoints such as EFS. Determining the place of ASCT in the treatment strategy for patients with HR-NB, it should be well understood that it most likely plays the role of a concomitant therapy aimed at restoring hematopoiesis after high-dose chemotherapy, rather than a part of antitumor treatment. However, an opportunity of antitumor function of natural killers within the hematopoietic stem cell transplant was noted in a few studies [52]. In most cases, the biomedical product for stem cell transplantation includes not only isolated CD34+ hematopoietic cells, which represent a minor subset of the cell transplant, but also mononuclear leukocytes, most of which are represented by lymphocytes. It is likely that the use of G-CSF for the mobilization of peripheral hematopoietic stem cells may contribute to the activation of antitumor immune effectors in vivo. To date, the content and functional status of NK and T cells within the graft and their antitumor potential has not been practically evaluated during ASCT procedure. The existing controversial data require rethinking the strategy of high-dose chemotherapy and ASCT in patients with HR-NB taking into account the risk–benefit ratio and economic costs [53,54]. Most studies demonstrated the advantage of ASCT over standard chemotherapy. However, the studies were different in patient cohorts, induction therapies, types of substitution therapy, and treatment methods, which explains the differences in the clinical outcomes of the analyzed studies. In addition, an unambiguous assessment of the effectiveness of ASCT is complicated, since most of the data were received in the non-randomized trials. So far, only a few randomized controlled trials have been reported.

### 2.4. Adoptive Immunotherapy

#### 2.4.1. NK Cells

NK cells are effector cells of the innate immunity that play a key role in antitumor immunological surveillance [55]. Solid tumors in children, including NB, have low immunogenicity because of low expression of the main histocompatibility complex I (MHC-I) and little mutational burden [56]. NK cells implement their killer activity against tumor cells in an antigen/MHC-independent manner and, unlike cytotoxic T-lymphocytes, can recognize and eliminate NB cells. In addition, NKs mediate antibody-dependent cellular cytotoxicity in the course of targeted monoclonal antibody-based (mAbs) therapy. Anti-GD2 mAbs, along with cytokine therapy (including IL-2 and GM-CSF), is a promising area in the treatment of HR-NB. Cytokine therapy promotes NK activation in vivo and, obviously, mediates antibody-dependent cell-mediated cytotoxicity (ADCC) in combination with Anti-GD2 mAbs.

A prospective single-arm phase II clinical trial evaluated the effectiveness of combined chemoimmunotherapy in children with HR-NB. Sixty-two patients received six cycles of induction chemotherapy in combination with anti-GD2 mAbs (hu14.18K322A), granulocyte–macrophage colony-stimulating factor (GM-CSF) and low doses of interleukin-2 (IL-2). Sixty patients (97%) had a partial response. None of the patients had any disease progression during the induction phase. The three-year event-free survival (EFS) reached 73.7%, and OS was 86.0% [57].

NK cells can improve survival outcomes through direct cytotoxic effects on tumor cells and through NK cell-mediated ADCC [58]. Various sources are used for NK cell therapy: autologous and allogeneic NK cells isolated from peripheral blood apheresis products or obtained from umbilical cord blood [59]. In experiments on the HR-NB mouse model, adoptive immunotherapy with activated NK cells in combination with dinutuximab showed a significant increase in animal survival [60]. In a Phase I clinical trial, four patients with HR-NB received adoptive immunotherapy with activated NK cells (1–5 × 10^7^ cells/kg) after consolidation and ASCT. Two patients achieved complete remission within 9–18 months. One patient registered a partial effect and one patient developed disease progression. Despite a small size of the patient cohort and the non-randomized study, the authors reported a favorable safety profile of allogeneic NK cells (1–59.5 × 10^6^ CD3-/CD56+ cells/kg) used in combination with anti-GD2 mAbs and IL-2, and noted the potential of the strategy as a neoadjuvant therapy in patients with HR-NB [61].

In a pilot study, five patients with NB received haploidentical NK cells after lymphodepletion with the subsequent ASCT. All patients achieved successful neutrophil and platelet engraftment. No adverse reactions were observed during and after NK cell infusion. The study showed acceptable tolerance of including NK cell infusion before ASCT as a component of a conditioning phase for consolidation therapy in children with HR-NB [62]. A phase II clinical trial involving 62 patients with HR-NB evaluated the kinetics, phenotype, and functions of NK cells throughout the multimodal treatment, including ASCT and subsequent NK cell therapy at a dose of 25 × 10^6^ cells/kg. Temporary engraftment was observed in all patients; however, the authors could not show that the engraftment of NK cells led to the improved clinical effect [63]. Kanold et al. [64] described a case of treating relapses developing 22 months after haplo-ASCT in a six-year-old child with HR-NB. Complete remission was achieved with combined chemoimmunotherapy using allogeneic NK cells, IL-2, and temozolomide/topotecan.

Talleur et al. performed a phase II study involving 30 patients with HR-NB who received a consolidation therapy with experimental immunotherapy. The consolidation phase included a busulfan/melphalan (Bu/Mel) conditioning regimen, autologous hematopoietic cell transplant (AHCT), immunotherapy with hu14.18K322A (anti-GD2 mAb), GM-CSF, IL-2, and adoptive transfer of haploidentical NKs. The authors registered grade 3/4 adverse events (AE), but no 5 grade AE. Eight patients had veno-occlusive disease (VOD) and 3 had VOD with mild multi-organ dysfunction, which were well managed and resulted in complete resolution. Other toxicities were similar to those previously reported with AHCT with Bu/Mel conditioning. Regarding tolerance of NK infusions, no acute graft-versus-host disease or additional toxicities were observed as compared to those patients who did not receive NK transfers. Taking into account the intensive multimodal regimen for HR-NB treatment and the need for improved therapies, the authors reported good tolerance of AHCT and adequate hematologic recovery after the procedure. The occurrences of VOD in patients who received NKs were similar to those patients who had no NK transfers. Thus, the authors confirmed that NK therapy showed no increased adverse events, demonstrated the feasibility of the consolidation regimen, and had an acceptable acute toxicity profile [65].

Another pilot study evaluated the safety and efficacy of anti-GD2 mAbs (hu14.18K322A) in combination with chemotherapy, cytokines, and haploidentical NK cells in 13 patients with HR-NB. The study demonstrated no NK-therapy-associated adverse events. The objective response was 61.5% with a CR/VGPR (complete response/very good partial response) rate of 38.5%. The median time to progression was 274 days and the 1-year survival rate achieved 77% [66].

Adoptive cell therapy (ACT) with the ex vivo activated NK cells is considered as a promising immunotherapeutic approach for improving the treatment effectiveness in children with HR-NB [67]. However, the presented data suggest a conclusion only about the safety of NK therapy, while the effectiveness of the adoptive immunotherapy requires more evidence. To date, no randomized clinical trials were registered to evaluate the feasibility of including NK cells in the multimodal treatment strategy for patients with HR-NB. In the future, it is necessary to determine the range of effective doses of NK cells and the place of NK therapy in the treatment of patients with HR-NB.

#### 2.4.2. CAR Lymphocytes

The technology for generating genetically modified lymphocytes with a chimeric T cell receptor (CAR) has been rapidly developing in the last decade. Anti-CD19-CAR-T therapy revolutionized the treatment of B cell hemoblastoses [68]. However, the effectiveness of CAR-T therapy in solid tumors has not yet shown high efficacy. The success of the first CAR-T cell therapies initiated the design of new variants of CAR-T cells targeting various tumor-associated antigens, including glycolipid antigen GD2 expressed on the tumor cells of neuroectodermal origin, in particular, on NB cells [69]. A later study of CAR-(EBV)-specific T cells in 19 patients with HR-NB achieved the following: eight patients were in remission and three had complete response (CR). No severe or dose-limiting toxicities or no neurological toxicity were detected. Three patients had localized pain of grades 1–3 [70]. GD2 CAR-T cells of the third generation achieved an antitumor effect, but the effect was transient and it was similar to that of GD2 CAR-T cells of the first generation. In a Phase I clinical trial involving 12 children (male with a median age of 4.5 years, range 1.8–7 years) with R/R NB (relapse/refractory NB), second-generation GD2 CAR-T cells with the CD28/CD3 signaling domain demonstrated safety and absence of non-tumor toxicity in NB patients. However, a partial effect in the form of regression of soft tissue and bone marrow lesions was observed in three out of six patients who received the maximum dose of GD2 CAR-T cells (10^8^ CAR-T cells/m^2^) [71].

A single-group Phase I study of 4SCAR-GD2 T cell treatment included 10 children with HR-NB. Before the introduction of 4SCAR-GD2 T cells, patients received fludarabine and cyclophosphamide for lymphodepletion. 4SCAR-GD2 T cells were infused one to three times with an interval of 3–6 months. The study demonstrated that the 4SCAR-GD2 T cells persisted for more than 6 months after infusion. Four patients achieved disease stabilization within 1 year and OS over 4 years [72].

A recent Phase I clinical study evaluated the efficacy of the first-generation GD2-CAR-T cells in eleven patients under 21 years old with HR-NB; three of them achieved a CR, which remained stable in two patients: one for 8 years and the other for more than 18 years. Five of the other eight patients, who had no signs of the disease at the time of CAR-T cell therapy, had an event-free period of 10–15 years. Thus, the use of first-generation vectors achieved a long-term disease control in patients with NB after GD2 CAR-T cell therapy [73].

Another study enrolled nineteen patients with NB, including eleven with relapses and eight patients with no signs of the disease, though five of them had a history of relapses, and three received standard HD treatment. After GD2 CAR-T cell therapy, three out of eleven patients had a CR, and one had a partial response. One of the patients with the CR subsequently relapsed, but the other two had a response that persisted: one—for 8 years until the patient was not available for the follow-up, and the other—for over 18 years. The EFS after 15 years was 31.6%, and the OS reached 36.8% [74].

Natural killer T cells (NKT) in the peripheral blood of cancer patients play an important role in the antitumor response, and a favorable outcome seems to be associated with a high number of NKT cells. The use of NKT cells has become interesting for the rapidly developing CAR lymphocyte technology [75]. A Phase I study including 12 children with NB evaluated GD2-CAR NKT therapy. The main objectives were safety and the determination of the maximum tolerated dose (MTD). The antitumor activity of GD2-CAR NKT was evaluated as a secondary endpoint. No dose-limiting toxicity was observed; one patient had grade 2 cytokine release syndrome, which was treated with tocilizumab. The objective response rate (ORR) was 25%, including two partial responses and one CR. The authors made a conclusion that GD2-CAR NKT therapy was safe and could mediate ORR in patients with NB [76]. In a Phase I/II study, 27 children with R/R HR-NB received autologous GD2-CAR-T cells of the third generation expressing the inducible caspase 9 suicide gene. Six weeks after GD2-CAR-T cell infusion, nine (33%) of patients achieved a CR. At the median follow-up period of 1.7 years, five out of nine patients had a CR, two patients had relapses and received further infusions, two patients died [77].

So far, clinical studies have shown the potential therapeutic activity of GD2 CAR-T cells in patients with low tumor burden. Obviously, the insufficient efficacy of GD2 CAR-T cells in HR-NB is determined by the heterogeneity of the tumor and the selection of GD2- cell clones evading specific immunotherapy. It should be noted that to date, the published data refer to small and non-randomized clinical trials only, which do not allow us to unambiguously assess the effectiveness of CAR-T and CAR-NKT therapy for HR-NB. Significant differences in clinical results were determined by the different variants and generations of CAR-T cells and a fairly wide range of therapeutic doses from 1 × 10^6^ to 1 × 10^9^ cells/kg. In addition, certain difficulties in comparing the results of clinical trials of CAR-T and CAR-NKT therapy are associated with the inclusion of patients with different NB stages, previous therapies, and combined treatments (Table 1).

One of the promising directions for increasing the efficiency of CAR lymphocytes is the design of tandem or multi-modular CARs targeting various NB-associated antigens. Another option for improving CAR therapeutic functions suggests the development of a universal CAR, where the antigen-recognizing site is replaced by CD16 receptor with high affinity that determines ADCC. Such universal CAR-T cells can be designed to target various tumor cell clones by combining with monoclonal antibodies. An important and still unresolved problem is the identification of the place of CAR-T therapy in the treatment of HR-NB, which limits a wider use of this strategy.

#### 2.4.3. TIL Therapy

Tumor-infiltrating lymphocytes (TIL) are found in most solid tumors; therefore, they can be used for immunotherapy. In particular, a clinical study demonstrated an objective response in over 50% of patients with advanced melanoma after treatment with activated and expanded ex vivo TILs in combination with high doses of IL-2. The studies demonstrated that TIL therapy had therapeutic potential against a number of solid tumors. In particular, in patients with head and neck squamous cell carcinoma, non-small cell lung cancer, cervical cancer, and ovarian cancer, the ORR was 55.6% and partial response was 44.4%, and disease control achieved 77.8% [78].

The NB microenvironment predominantly consists of anti-inflammatory immune cells such as tumor-associated macrophages (TAM) and myeloid suppressor cells (MDSC), which limit TIL therapy effectiveness in this population of patients [79]. Interestingly, T-lymphocytes were found in the NB microenvironment [80]. Ollé Hurtado et al. [81] managed to isolate T-lymphocytes from the removed NB samples and achieved their expansion in vitro. The expanded TILs effectively destroyed the cells of the transplanted tumor cell lines in vitro, but were practically useless against autologous NB cells. The authors also showed that activated TILs could be successfully modified by viral vector transduction, and the generated CAR-TILs had high cytotoxic activity against NB cell lines. Despite encouraging experimental results, so far, no data are available on the clinical use of TILs in NB patients, and the perspectives for this therapeutic strategy require preclinical confirmation. An important factor limiting the technology for NB treatment is the lack of killer activity of the activated TILs against autologous tumor cells. Obviously, the cells forming the tumor have gone through all the stages of immune editing and acquired the state evading the host’s immunity. Another negative factor for TIL therapy is low NB immunogenicity, resulting primarily from the low MHC expression, which blocks T killer antitumor functions, even in the presence of a tumor antigen. CAR-TILs can overcome this barrier due to the MHC-independent cytotoxicity towards targeted tumor cells. However, the question arises as to whether CAR-TILs have any advantage over classical CAR-T cells. In addition, it is necessary to remember that TIL-based treatment should be combined with several cycles of high-dose IL-2 therapy, which is associated with marked adverse events [82].

## 3. Bioinformatics and Neuro-Antigen Discovery in the Tumor Microenvironment of Neuroblastoma

NB is a pediatric malignancy arising from neural crest-derived sympathoadrenal progenitors and remains one of the leading causes of cancer-related mortality in children worldwide [83,84]. The clinical outcomes for high-risk NB patients remain suboptimal despite aggressive multimodal therapy including chemotherapy, radiotherapy, autologous stem cell transplantation, and immunotherapy. This has driven an intensified focus on deciphering the TME and identifying tumor-specific targets to enable effective immunotherapeutic interventions [32].

### 3.1. The Neuroblastoma Tumor Microenvironment and Immune Landscape

The NB TME is a complex and dynamic ecosystem comprising tumor cells, cancer-associated fibroblasts, immune infiltrates (such as CD8^+^ T cells, regulatory T cells, and myeloid-derived suppressor cells), vasculature, and ECM components. Unlike many adult solid tumors, NB typically demonstrates a relatively immune-cold phenotype with sparse effector T cell infiltration and dominant immunosuppressive populations [85]. One critical barrier is the dense ECM, particularly fibrillar collagens such as types I and XI, which increase tumor stiffness and impede T cell trafficking. Experimental interventions such as collagen-targeted thermal ablation have shown promise in softening the ECM, reducing lysyl oxidase (LOX) activity, and enhancing immune cell access [31,32]. ECM plays a crucial role in modulating antitumor immunity. In particular, preclinical and a few clinical studies showed that selective inhibition of functional components of cell–ECM interaction, such as hyaluronic acid (HA), matrix metalloproteinases (MMPs), and integrins could suppress tumor progression and enhance the efficacy of ICI treatment, chemotherapy, or immunotherapy in melanoma [86].

### 3.2. Neuro-Antigens: Developmentally Restricted Tumor Targets

A hallmark of NB is its low tumor mutational burden (TMB), resulting in a paucity of classical neoantigens derived from somatic mutations [87]. However, NB reactivates a suite of embryonic neural crest-related proteins known as “neuro-antigens”, tumor-associated antigens (TAAs) that are minimally expressed in postnatal tissues but highly expressed in NB tumors [88]. These include transcription factors (e.g., PHOX2B), enzymes involved in catecholamine synthesis (e.g., tyrosine hydroxylase, TH), membrane glycosylation enzymes (e.g., GD2 synthase), and other lineage-specific proteins (e.g., GAP43, ALDH1A2) [89,90,91]. Their restricted expression pattern and immunogenic potential make them attractive targets for vaccines, T cell receptor (TCR) therapies, and CAR-T cell development. A selection of representative neuro-antigens with therapeutic relevance is presented in Table 2.

### 3.3. Immunoinformatics Tools for Epitope Discovery

Immunoinformatics has become essential in the systematic identification of immunogenic epitopes within neuro-antigens. Tools such as NetMHCpan 4.0 and MHCflurry 2.0 utilize machine learning and mass spectrometry-trained models to predict peptide binding to major histocompatibility complex (MHC) molecules across diverse human leukocyte antigen (HLA) genotypes [92,93]. The Immune Epitope Database (IEDB) 3.0 provides a comprehensive platform integrating multiple predictors for antigen processing, MHC binding, and epitope clustering [94]. Motif-based methods like SYFPEITHI offer complementary approaches [95].

Other tools include VaxiJen 2.0 for alignment-free antigenicity prediction [96], AllerTOP v.2 for allergenicity classification [97], and PickPocket for structure-based binding affinity estimation [98]. Importantly, population coverage analysis from IEDB enables selection of epitopes likely to elicit immune responses across diverse ethnic groups [94]. Table 3 provides an overview of key bioinformatics tools commonly used for predicting neuro-antigen epitopes and evaluating their immunogenic, allergenic, and population-specific potential.

### 3.4. AI and Multi-Omics in Epitope Prioritization

To enhance the prediction of clinically relevant neoantigens, artificial intelligence (AI) and multi-omics data integration are being increasingly adopted. Single-cell RNA sequencing (scRNA-seq), immunopeptidomics, and spatial transcriptomics enable high-resolution mapping of antigen expression and presentation across tumor and immune cell subsets [99,100].

AI-powered models such as DeepHLApan, MHCnuggets, and EPIC integrate deep neural networks, long short-term memory (LSTM) architectures, and transcriptomic data to improve binding and immunogenicity prediction [101,102,103]. DeepVacPred evaluates physicochemical features to rank epitopes [104], while CancerEpitopeAI combines gene expression, antigen processing, and structural features to identify prioritized targets [105]. Tools like scANNA apply transformer-based models to scRNA-seq data for high-fidelity mapping of antigen presentation networks [106]. Table 4 presents an overview of cutting-edge algorithms that combine artificial intelligence and high-dimensional omics profiling in NB immunogenomics. Prediction of clinically relevant neoantigens in neuroblastoma aims to identify highly immunogenic tumor-specific peptides in samples with low mutational burden for the development of personalized vaccines and improved immunotherapy response, particularly in high-risk diseases. Prediction processes include exome/RNA sequencing to identify somatic mutations (e.g., ALK) and machine learning (e.g., NetMHCpan) to estimate HLA binding, expression, and T cell recognition. These approaches are currently used to design individual antitumor vaccines [107,108].

## 4. Future Directions

The integration of bioinformatics and immunogenomics has substantially advanced our ability to identify actionable antigens in NB. Although the low TMB poses a challenge, the presence of neuro-antigens and advances in AI-guided, multi-omics-integrated platforms offer significant opportunities. Future research should prioritize the functional validation of predicted epitopes through human T cell assays, organoid systems, and in vivo tumor models. Additionally, patient-specific HLA typing and immune deconvolution will be essential to enable personalized vaccine and T cell therapy strategies in HR-NB.

## 5. Conclusions

Cell-based technologies have a high potential for the treatment of patients with HR-NB. In particular, a number of clinical trials have studied ASCT, NK cells, CAR-T, and CAR-NKT cells in the adoptive therapy for HR-NB. However, the results are often controversial due to the lack of large randomized trials done using replicated treatments and, so far, it seems impossible to make a distinct conclusion regarding the effectiveness of each type of the adoptive therapy. Hopefully, further randomized clinical trials will help determine the role of cell-based technologies in the multimodal treatment of patients with HR-NB.

## Figures and Tables

**Table 1:** Representative studies of the adoptive cell immunotherapy for HR-NB and R/R NB.

Study, Cells, Ref.	Patients, n	Diagnosis	Additional Therapy	Response
Phase I. GD2-CAR-T cells, 1.2 × 10^7^–3.1 × 10^8^ cells/m^2^ [70]	19	HR-NB	EBV-specific CTL	OS: 931 days CR: 16%.
Phase I. GD2-CAR-T cells, 10^8^ cells/m^2^ [71]	12	R/R NB	N/A	No ORR
Phase I. 4SCAR-GD2 T cell, 0.13–34 × 10^6^ cells/kg [72]	10	R/R NB	N/A	6: SD at 6 mon 4: SD at 1 year OS: 25 mon PFS: 8 mon
Phase I. GD2-CAR-T cells, 2 × 10^7^ cells/m^2^ [73]	11	HR-NB	EBV-specific T-lymphocytes	3: CR at 8 mon
Phase I. GD2-CAR NKT, 3 × 10^6^–1 × 10^9^ cells/kg [76]	12	R/R NB	N/A	ORR: 25%
Phase I/II. GD2-CART01 expressing the inducible caspase 9 suicide gene. 3–10 × 10^6^ cells/kg [77]	27	R/R NB	N/A	ORR: 63% CR: 9 pts PR: 8pts
Phase I. Allogeneic NK, 1 and 5 × 10^7^ cells/kg [61]	4	HR-NB	auto-HSCT	>20 mon
Activated haploidentical natural killer cells. Phase I/II pilot study, 1 × 10^6^ CD56+ cells/kg [62]	5	HR-NB	auto-HSCT, High-activity (8.8–14.2 mCi/kg) 131I-MIBG	6 mon after auto-HSCT, CR: all patients
Pilot trial. Humanized Anti-GD2 mAb with chemotherapy and NK, 4.7 × 10^6^ to 59.5 × 10^6^ CD56+ cells/kg [66]	13	R/R NB	Anti-GD2 mAb with chemotherapy	ORR: 61.5%, CR + VGPR: 38.5%
Phase I. Adoptive immunotherapy with haploidentical NK and Anti-GD2 mAb, 1 × 10^6^–50 × 10^6^ CD3-CD56+ cells/kg [65]	30	R/R NB	Anti-GD2 mAb	CR + PR: 29%

Note: HR-NB—high-risk neuroblastoma, R/R NB—relapse/refractory neuroblastoma, OS—overall survival, CR—complete response, PFS—progress free survival, SD—stable disease, EBV—Epstein–Barr virus, CTL—cytotoxic T-lymphocytes, VGPR—very good partial response, NK—natural killer cells, mAb—monoclonal antibody, HSCT—hematopoietic stem cell transplantation, mon—months, N/A—not available.

**Table 2:** Representative neuro-antigens in neuroblastoma.

Antigen	Function	Expression in NB	Therapeutic Potential	Reference
PHOX2B	Transcription factor	High	Vaccine, TCR therapy	[89]
TH	Catecholamine biosynthesis	High	Immune marker	[90]
GD2 synthase	Ganglioside GD2 biosynthesis	High	CAR-T therapy	[90]
GAP43	Axonal growth cone marker	Moderate	Peptide-based immunotherapy	[91]
ALDH1A2	Retinoic acid biosynthesis	Moderate	Neoantigen candidate	[91]

**Table 3:** Bioinformatics tools for neuro-antigen epitope prediction.

Tool	Main Function	Target MHC Class	Strengths	Reference
NetMHCpan 4.0	Predicts peptide–HLA binding affinity	I, II	Pan-specific, MS data-integrated, high accuracy	[92]
MHCflurry 2.0	Machine learning epitope prediction	I	Combines presentation and processing model	[93]
IEDB Tools 3.0	Epitope binding, processing, clustering	I, II	Broadly validated, widely used, integrates multiple methods	[94]
SYFPEITHI	Motif-based epitope prediction	I	Simple interface, motif-based approach	[95]
VaxiJen 2.0	Antigenicity prediction (alignment-free)	N/A	Fast, good for tumor antigen screening	[96]
AllerTOP v.2	Allergenicity classification based on auto-cross covariance	N/A	Supports filtering potential allergens	[97]
PickPocket	Binding affinity prediction using pocket similarity	I	Works with limited data, complement to NetMHCpan	[98]
IEDB Population Coverage Tool 3.0	Population-specific HLA coverage analysis	I, II	Key for evaluating candidate global applicability	[94]

MHC class I, MHC class II, N/A= not available.

**Table 4:** AI and multi-omics frameworks in neuroblastoma antigen discovery.

Framework	Key Features	Application in Neuroblastoma	Reference
DeepHLApan	Deep learning for MHC binding	Epitope ranking across HLAs	[101]
MHCnuggets	LSTM model for class I/II prediction	Tumor antigen prioritization	[102]
EPIC	Expression-integrated epitope predictor	Immunogenicity scoring	[103]
DeepVacPred	Immunogenicity + physiochemical encoding	Neoantigen candidate selection	[104]
scANNA	AI-enabled single-cell antigen presentation	TME-specific antigen mapping	[106]

## Data Availability

No new data were created or analyzed in this study. Data sharing is not applicable to this article.

## Abbreviations

The following abbreviations are used in this manuscript:

ABMT	Autologous bone marrow transplantation
ADCC	Cell-mediated cytotoxicity
AML	Acute myeloid leukemia
ASCT	Autologous stem cell transplantation
CAF	Cancer-associated fibroblasts
CAR	Chimeric T cell receptor
CR	Complete response
DB	Dinutuximab beta
DC	Dendritic cell
EBV	Epstein–Barr virus
ECM	Extracellular matrix
EFS	Event-free survival
HIF	Hypoxia-inducible factor
GM-CSF	Granulocyte–macrophage colony-stimulating factor
GD2	Disialoganglioside
HR-NB	High-risk neuroblastoma
haplo-HSCT	Haploidentical hematopoietic stem cell transplantation
IL-2	Interleukin-2
MDSC	Myeloid-derived suppressor cells
MHC-I	Main histocompatibility complex I
MTD	Maximum tolerated dose
mAb	Monoclonal antibody-based
NB	Neuroblastoma
NK	Natural killer
NKT	Natural killer T cells
OS	Overall survival
TAM	Tumor-associated macrophage
TIL	Tumor-infiltrating lymphocytes
TGF	Tumor growth factor
TME	Tumor microenvironment
Treg	Regulatory T cells
VEGF	Vascular endothelial growth factor
